# New Appliances and Things Medical

**Published:** 1896-10-17

**Authors:** 


### NEW APPLIANCES AND THINGS MEDICAL.

### PALE CRYSTAL ALE.

(Michell, Goodman, Young, and Co., Limited, Stamford Hill Brewery.)

We have received a sample of the above ale, brewed and bottled by Messrs. Michell, Goodman, Young, and Co. From tests to which we have submitted it, we can detect no deleterious preservative or other objectionable chemical ingredient. It is a remarkably clear, sparkling beverage, with an agreeable flavour and aroma of hops, and we have no hesitation in recommending it as a light dinner ale which has been carefully and skilfully brewed.

